# Clinical evaluation of the i-gel Plus supraglottic airway in Japanese patients: A prospective observational study

**DOI:** 10.1371/journal.pone.0349108

**Published:** 2026-05-07

**Authors:** Toshiyuki Nakanishi, Ayaka Tani, Kei Matsumoto, Atsushi Ishikawa, Yuki Takami, Yuji Kamimura, Rina Kato, MinHye So, Kazuya Sobue

**Affiliations:** Department of Anesthesiology and Intensive Care Medicine, Nagoya City University Graduate School of Medical Sciences, Nagoya, Japan; Shanghai Jiao Tong University, CHINA

## Abstract

**Background:**

The i-gel® Plus, a new supraglottic airway, has a high oropharyngeal leak pressure of 32 ± 7 cmH_2_O in the European population. Because the i-gel® series was developed based on the laryngeal anatomy in Western populations, its performance in other regions, such as East Asia, is unknown. Therefore, we aimed to perform an initial clinical evaluation of i-gel® Plus in Japanese patients.

**Methods:**

This prospective observational study, conducted from March to June 2024 at a university hospital in Japan, recruited a total of 64 adult patients, 16 each of nonelderly (18–69 years) male, nonelderly female, elderly (≥ 70 years) male, and elderly female patients. Patients with a suspected high risk of aspiration or a predicted difficult airway were excluded. We recorded the oropharyngeal leak pressure (primary outcome), first-attempt success rate, and fiberoptic views of the i-gel® Plus. Continuous variables were evaluated for normality using the Shapiro–Wilk test and Q-Q plot. The groups were stratified by age and sex for exploratory analysis.

**Results:**

Of the 67 enrolled patients, three were excluded: one 70-year-old male because of failed ventilation and two because of missing data. Finally, 64 patients were analyzed, with 16 patients in each age-sex group. The oropharyngeal leak pressure was 23.7 ± 6.9 (mean ± standard deviation) cmH_2_O, which was normally distributed based on the Shapiro–Wilk normality test (P = 0.593) and Q-Q plot. The first-attempt success rate was 92% (including one failed ventilation case), and visible vocal cords were observed in 80% of cases. In exploratory comparisons by sex, male patients had lower oropharyngeal leak pressure and fewer visible vocal cords than female patients.

**Conclusions:**

The i-gel® Plus showed a high first-attempt success rate of 92%; however, its oropharyngeal leak pressure was lower than expected at 23.7 ± 6.9 cmH_2_O in this Japanese cohort.

## Introduction

i-gel® (Intersurgical, Wokingham, UK), a second-generation supraglottic airway (SGA) introduced in the 2000s [1,2], is characterized by its gel-like, noninflatable cuff designed based on laryngeal anatomy and is equipped with a gastric tube channel. It has a high first-time success rate and faster insertion time and is easy to place even for novices [3–5]. Therefore, i-gel® has been widely used for managing the airway during general anesthesia, securing the airway during resuscitation [6], and rescuing difficult airway situations [7]. Recent difficult airway management guidelines also emphasize the role of SGAs as rescue devices and as part of difficult airway management strategies [8–10].

The oropharyngeal leak pressure (OLP) serves as a key performance indicator of SGAs, reflecting the sealing pressure during positive pressure ventilation [11,12]. A high OLP is critical for evaluating the suitability of SGAs, particularly for advanced indications, such as in patients who are obese or require laparoscopic surgeries. Despite the favorable properties of i-gel® for its easy insertion, several meta-analyses have reported that i-gel® has lower OLP than other second-generation SGAs, such as LMA® ProSeal™, LMA® Protector™, and Ambu® AuraGain™ [13–15].

Recently, i-gel® Plus (Intersurgical) has been released, which has a larger gastric tube channel and a modified cuff shape to improve the sealing pressure, an advancement over the conventional i-gel®. Thus, i-gel® Plus may be useful for advanced SGA applications, such as use in patients with obesity or those requiring laparoscopic surgery. In the European multicenter study conducted by Werner et al. involving 1,000 patients, i-gel® Plus was reported to have a high OLP of 32 ± 7 cmH_2_O [16]. Because the i-gel® series was developed according to laryngeal anatomy in Europe and the USA [2], whether i-gel® Plus achieves the same high OLP in other regions, such as East Asia, where population anatomy may differ, is unknown [17]. Moreover, anatomical differences across populations may affect the positioning of the epiglottis.

For future studies and clinical practice, the generalizability of i-gel® Plus in different regions must be clarified. Therefore, this study aimed to collect data on i-gel® Plus in elderly and nonelderly male and female patients for initial clinical evaluation in a Japanese cohort.

## Materials and methods

The protocol of this study was approved by the Nagoya City University Graduate School of Medical Sciences and the Nagoya City University Hospital Institutional Review Board (60-23-0139, February 15, 2024) and registered at the Japan Registry of Clinical Trials (jRCT1042230158; Principal investigator, Toshiyuki Nakanishi; Date of registration, February 27, 2024). Written informed consent was obtained from all participants prior to study enrollment. The manuscript was written following the STROBE statement [18]. This prospective, observational study was conducted in the operating rooms at a university hospital in Japan to collect the clinical characteristics of i-gel® Plus in adult patients undergoing noncardiac surgery. The first patient was recruited on March 4, 2024, and the last was followed up until June 4, 2024.

Patients aged ≥ 18 years who were scheduled to undergo noncardiac surgery (except for thoracic surgery, head and neck surgery, surgery performed in the prone position, and surgery with a scheduled duration of ≥ 4 h) under general anesthesia were included. Patients were excluded if they were pregnant, had impaired consciousness, were suspected to have not fasted, had a high risk of gastroesophageal reflux or aspiration (symptomatic gastroesophageal reflux disease, intestinal obstruction, hiatal hernia, after esophagectomy, after total gastrectomy, or body mass index [BMI] ≥ 35 kg/m^2^), had airway abnormalities (anatomical abnormalities, occupying lesions, abscesses, trauma, or a history of surgery or radiation in the oral cavity, pharynx, larynx, or trachea), had a predicted difficult airway (mouth opening < 2.5 cm, impossible neck movement, or a history of difficult airway management), or had noticeable hoarseness or sore throat preoperatively.

The eligibility was initially assessed using electronic medical records, followed by preoperative evaluation that included airway examination and assessment of throat symptoms. All participants were followed until postoperative day 1. Given that the fitting performance of SGAs may differ depending on age and sex [4,19], this exploratory study, which aimed to collect the first clinical data for i-gel® Plus in the East Asian population, recruited 16 each of nonelderly (18–69 years) male, nonelderly female, elderly (≥ 70 years) male, and elderly female patients.

To avoid failure by inexperienced anesthesiologists, only anesthesiologists who had experience with at least 20 cases of conventional i-gel® participated in this study. Before study participation, all attending anesthesiologists were required to perform three successful placements of size 3 i-gel® Plus with a manikin.

After the patient entered the operating room, electrocardiogram, peripheral oxygen saturation, noninvasive blood pressure, and bispectral index (BIS) monitoring commenced. Then, a peripheral venous line was secured, and video recordings were started, including the patient’s head and vital sign monitor, for precise and objective measurement. After 3 min of oxygenation with 100% oxygen at 6 L/min, anesthesia was induced with intravenous anesthetics. The anesthetic type and volume and the decision to use neuromuscular blocking drugs were left to the attending anesthesiologists.

Based on the literature and our clinical experience, the size of i-gel® Plus was selected primarily by sex (size 3 for females and size 4 for males) because previous studies suggested that sex-based sizing may improve device fit in some settings [20,21]. However, the attending anesthesiologist was allowed to make the final size selection based on the manufacturer’s weight criteria (size 3 for patients weighing 30–60 kg and size 4 for 50–90 kg) when judged clinically appropriate. The back, sides, and tip of the i-gel® Plus body were lubricated with water-soluble jelly. The attending anesthesiologist inserted i-gel® Plus after confirming a sufficient anesthesia level (BIS < 60 and absence of resistance to mouth opening). During i-gel® Plus insertion, whether successful or not, the number of attempts, insertion time, subjective difficulty, and airway injury were recorded.

Successful placement was defined as achieving mechanical ventilation of at least 5 mL/kg of the tidal volume through i-gel® Plus. The number of attempts was increased if the procedure was interrupted, and mask ventilation was performed if the placement failed or if there was a change in the anesthesiologist who performed the procedure. The insertion time was measured from when i-gel® Plus was picked up until the first upslope appeared in the capnogram, consistent with the definition used in several previous studies [3,5,22]. Subjective difficulty was rated on a three-point scale as easy, not easy, or difficult.

After successful i-gel® placement, the bubble test was performed. This test was deemed positive if air leaks occurred, and the jelly sealed in the gastric tube channel was blown out [12]. If an air leak or the bubble test was positive, the position of i-gel® Plus was adjusted to improve the fit. If sufficient mandatory ventilation (≥ 5 mL/kg of the tidal volume) with clinically acceptable air leaks (≤ 6 L/min of the fresh gas flow) could not be obtained even after repositioning i-gel® Plus more than twice, the patient’s trachea was intubated and dropped from the study. Thereafter, the attending anesthesiologist managed the anesthesia at their discretion, and no further outcomes were obtained.

After the successful placement of i-gel® Plus, the well-lubricated 15-Fr gastric tube was inserted via the gastric tube channel. Whether the placement was successful or not, the number of attempts, insertion time, and subjective difficulty were evaluated during the gastric tube insertion. The gastric tube insertion was successful if gastric bubble sounds were auscultated with 10 mL of air. The insertion time was defined as the time from picking up the gastric tube to confirming the gastric bubble sounds.

Then, the OLP was measured. It was defined as the airway pressure at which the pressure stopped increasing or a leak occurred with a fresh gas flow of 5 L/min and a pressure limiting valve was set to 40 cmH_2_O [12].

Finally, the fiberoptic score was observed using a flexible bronchoscope through the ventilation port of i-gel® Plus. The fiberoptic score was graded as follows: 4, only vocal cords were seen; 3, vocal cords and posterior epiglottis were seen; 2, vocal cords and anterior epiglottis were seen; and 1, vocal cords were not seen but functioned adequately [23]. Visible vocal cords were defined as a fiberoptic score of 2–4. These aforementioned evaluations were performed preoperatively.

Intraoperatively, anesthesia was maintained using fentanyl, remifentanil, and propofol or sevoflurane. During anesthesia, flurbiprofen, acetaminophen, and dexamethasone were administered intravenously, if indicated. Postoperatively, i-gel® Plus was removed after confirming the patient’s consciousness. During i-gel® Plus insertion and after removal, airway injury was assessed using a standardized checklist. Visible injury to the lips, tongue, and teeth, as well as blood attachment on the i-gel® Plus, was systematically recorded for each patient. Postoperative hoarseness and sore throat were evaluated using a four-point scale in the postanesthesia care unit (PACU) and hospital ward the next morning after surgery [24]. Hoarseness was graded none, mild (noticed by patients), moderate (obvious to observers), and severe (dysphonia) [24]. Sore throat was graded as none, mild (pain with swallowing), moderate (constant pain increased by swallowing), and severe (pain interfering with eating or requiring analgesic medications) [24].

The primary outcome was an OLP. The secondary outcomes were i-gel® Plus placement-related outcomes (first-attempt success rate, overall success rate, number of attempts, insertion time, and subjective difficulty), gastric tube insertion-related outcomes (first-attempt success rate, overall success rate, number of attempts, insertion time, and subjective difficulty), performance tests (bubble test and fiberoptic score), insertion-related injuries (any bleeding or injury to the lips, tongue, pharynx, larynx, or teeth), blood attachment on i-gel® Plus after removal, and postoperative hoarseness and sore throat.

Patients’ age, sex, height, weight, BMI, American Society of Anesthesiologists physical status, Mallampati class, neck mobility, comorbidities, smoking history, surgery type, surgery time, and anesthesia time were also obtained from the electronic medical records and anesthesia information management system.

This study was primarily designed as an exploratory observational study to collect initial clinical data on i-gel® Plus in Japanese patients, rather than to formally test a specific hypothesis. We recruited 16 each of nonelderly male, nonelderly female, elderly male, and elderly female patients to ensure balanced representation across age and sex categories. This design allowed exploratory comparisons by age (32 nonelderly vs. 32 elderly patients) and by sex (32 male vs. 32 female patients), but the study was not specifically powered for definitive comparisons between these groups. Based on previous studies and our clinical experience, the standard deviation (SD) of the OLP for i-gel® Plus was estimated to be approximately 6 cmH_2_O [14]. When the clinically meaningful difference in the OLP was set at 5 cmH_2_O, 24 patients per group would provide an α of 0.05 and 1 − β of 0.8. We therefore planned the study to include 32 patients for each comparison by age and sex to improve the precision of these exploratory estimates. These exploratory comparisons were not adjusted for multiple comparisons.

Patients with missing outcome data were excluded from the analysis, and the main analyses were based on complete cases. Other missing data were not imputed and are described in the corresponding tables. Because missing outcome data were limited, no sensitivity analysis for missing data was performed. Because successful placement of the i-gel® Plus was itself a crucial outcome, patients with failed placement were included in the relevant placement-related analyses even if no further outcome data were available. Continuous variables were evaluated for normality using the Shapiro–Wilk test and visual inspection of Q-Q plots and histograms. Then, normally distributed data are expressed as mean ± SD, and non-normally distributed data are expressed as median (Q1, Q3). Categorical variables are expressed as numbers (%). In the exploratory comparisons between groups stratified by age and sex, Welch’s two-sample t-test for normally distributed continuous variables, Mann–Whitney U test for non-normally distributed continuous variables, and Fisher’s exact test for categorical variables were performed. All statistical analyses were performed using R 4.4.0 (R Foundation for Statistical Computing, Vienna, Austria).

## Results

**Fig 1:**
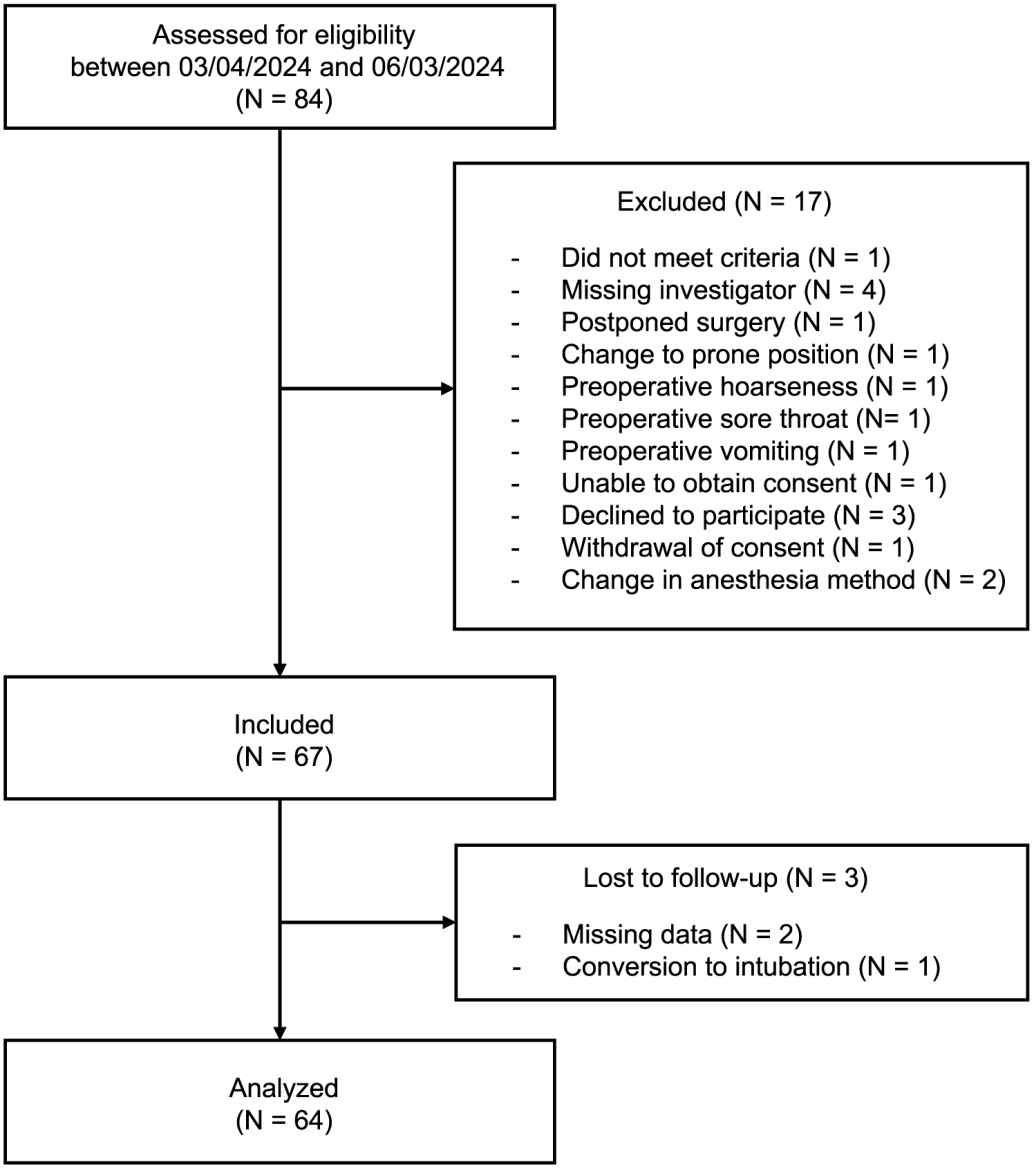
Patient flowchart.

In this study, 84 patients were assessed for eligibility, and 17 were excluded (Fig 1). Of the 67 patients included, two were dropped because of missing outcome data, and one 70-year-old male patient was dropped because of failed ventilation due to poor fit of the i-gel® Plus and subsequent conversion to tracheal intubation. Finally, 64 patients were analyzed, with 16 patients in each age-sex group, as previously determined. The background and perioperative characteristics of these patients are summarized in Tables 1, S1 and S2. Only one female patient used size 4 i-gel® Plus, deviating from the initial size selection based on sex.

**Table 1:** Background and perioperative characteristics of the patients.

Variables	Total N = 64
Age	69 (55, 77)
Male sex	32 (50)
Height (cm)	160.9 (152.0, 167.9)
Weight (kg)	60.8 (50.9, 68.1)
BMI (kg/m^2^)	23.2 (20.4, 26.3)
ASA classification, 1 / 2 / 3 / 4	11 (17) / 48 (75) / 4 (6) / 1 (2)
Mallampati class, 1 / 2 / 3 / Not recorded	50 (78) / 8 (13) / 2 (3) / 4 (6)
Restricted neck movement, None / Mild	60 (94) / 4 (6)
Induction agent, Propofol / Remimazolam	60 (94) / 4 (6)
Induction using neuromuscular blocking agent	60 (94)
Size of i-gel® Plus, 3 / 4	31 (48) / 33 (52)
Anesthesiologists with ≥ 2-year experience	51 (80)
Surgery time (min)	69.0 (53.3, 97.3)
Anesthesia time (min)	116.0 (96.5, 141.0)

The data are shown as median (Q1, Q3) or number (%).

ASA: American Society of Anesthesiologists, BMI: body mass index

**Fig 2:**
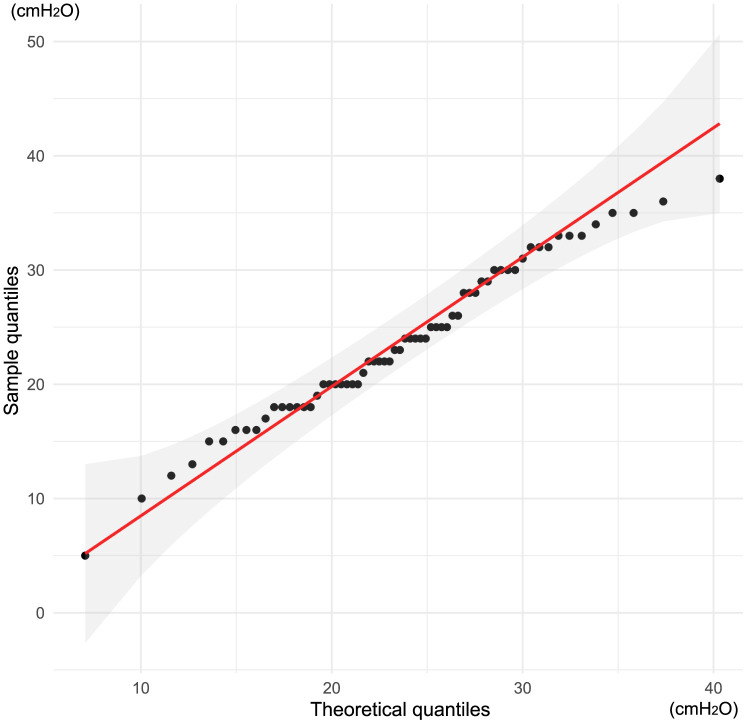
Q-Q plot for the oropharyngeal leak pressure of i-gel® Plus. The red line indicates the theoretical distribution when following a normal distribution, and the gray-shaded area shows its 95% confidence interval.

**Fig 3:**
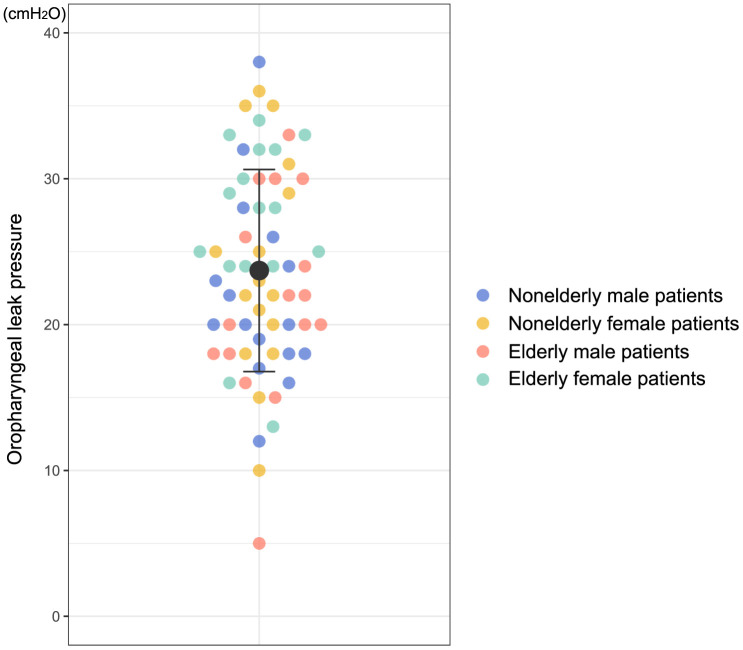
Distribution of the oropharyngeal leak pressure of i-gel® Plus.

The clinical performance of i-gel® Plus is shown in Table 2. The Shapiro–Wilk normality test (P = 0.593) and Q-Q plot showed that the OLP of i-gel® Plus was normally distributed (Fig 2). In a total cohort comprising 64 patients, the OLP of i-gel® Plus was 23.7 ± 6.9 cmH_2_O (Fig 3). Regarding outcomes related to i-gel® Plus insertion, the first-attempt success rate was 92% (including one failed ventilation case), the overall success rate was 98%, the insertion time was 23.0 (17.0, 31.8) s (P < 0.001 for Shapiro–Wilk normality test), and 81% of cases were rated as easy. The vocal cords were visible in 80% of cases.

**Table 2:** Clinical performance of i-gel® Plus in the total cohort.

Variables	N = 64
Oropharyngeal leak pressure (cmH_2_O)	23.7 ± 6.9
**i-gel® Plus placement**	
First-attempt / overall success^*^	60 (92) / 64 (98)
Number of attempts, 1 / 2 / 3	60 (94) / 2 (3) / 2 (3)
Insertion time (s)	23.0 (17.0, 31.8)
Difficulty, Easy / Not easy / Difficult	52 (81) / 11 (17) / 1 (2)
Device change	0 (0)
Audible leak	5 (8)
Positive bubble test	2 (3)
Fiberoptic score, 4 / 3 / 2 / 1	19 (30) / 17 (27) / 15 (23) / 13 (20)
**Gastric tube insertion**	
Number of attempts, 1 / 2	63 (98) / 1 (2)
Insertion time (s)	27.0 (22.8, 36.0)
Difficulty, Easy / Not easy / Difficult	60 (94) / 4 (6) / 0 (0)
Size change	1 (2)
**Injury during placement**	
Tooth / Lips / Tongue / Glottis	0 (0) / 1 (2) / 0 (0) / 0 (0)
Blood attachment	2 (3)
**Injury after removal**	
Tooth / Lips / Tongue	0 (0) / 2 (3) / 0 (0)
Blood attachment	1 (2)

The data are shown as mean ± standard deviation, median (Q1, Q3), or number (%).

* The success rate was evaluated for 65 patients, including one failed ventilation patient.

Regarding the gastric tube insertion, the first-attempt success rate was 98%, with a 100% success rate within two attempts. The insertion time was 27.0 (22.8, 36.0) s, and 94% of cases were rated as easy. Postoperative hoarseness was observed in 11 patients (17%) in the PACU and 5 patients (8%) on postoperative day 1 (S3 Table). Postoperative sore throat was observed in 6 patients (9%) in the PACU and 7 patients (11%) on postoperative day 1.

Exploratory comparisons of i-gel® Plus performance between nonelderly and elderly patients and between male and female patients are shown in Table 3 and S1 Fig. The OLP was lower in male patients than in female patients (21.9 ± 6.7 vs. 25.5 ± 6.8 cmH_2_O, mean difference [95% confidence interval, CI] −3.53 [−0.16 to −6.90], P = 0.040); however, the mean difference did not reach a clinically meaningful difference. In contrast, no difference was found in the OLP between the nonelderly and elderly patients (23.1 ± 7.0 vs. 24.3 ± 6.9 cmH_2_O, mean difference [95% CI] −1.28 [−4.76 to 2.19], P = 0.464). The first-attempt success rate, insertion time, and ease of placement were comparable between nonelderly and elderly patients and between the male and female patients (Table 3). The fiberoptic score was higher in elderly patients than in nonelderly patients and in female patients than in male patients. In particular, male patients had significantly fewer visible vocal cords than female patients (63% vs. 97%, relative risk [95% CI] 0.65 [0.49 to 0.85], P = 0.001). However, no difference was found between the nonelderly and elderly patients (72% vs. 88%, relative risk [95% CI] 0.82 [0.64 to 1.06], P = 0.213).

**Table 3:** Exploratory comparisons of the i-gel® Plus performance between nonelderly and elderly patients and between male and female patients.

Outcomes	Nonelderly N = 32	Elderly N = 32	Relative risk (95% CI)	P-value	Male N = 32	Female N = 32	Relative risk (95% CI)	P-value
OLP (cmH_2_O)	23.1 ± 7.0	24.3 ± 6.9	−1.28 (−4.76 to 2.19)^†^	0.464^‡^	21.9 ± 6.7	25.5 ± 6.8	−3.53 (−0.16 to −6.90)^†^	0.040^‡^
First-attempt success	32 (100)	28 (88)	1.14 (1.00 to 1.30)	0.113^§^	30 (94)	30 (94)	1.00 (0.88 to 1.13)	1^§^
Insertion time (s)	23.0 (17.0, 30.3)	24.0 (18.0, 34.3)		0.563^II^	23.0 (18.0, 29.3)	24.0 (16.0, 34.3)		0.995^II^
Easy placement	28 (88)	24 (75)	1.17 (0.92 to 1.48)	0.337^§^	28 (88)	24 (75)	1.17 (0.92 to 1.48)	0.337^§^
Fiberoptic score				0.040^§^				0.002^§^
4	5 (16)	14 (44)			6 (19)	13 (41)		
3	11 (34)	6 (19)			7 (22)	10 (31)		
2	7 (22)	8 (25)			7 (22)	8 (25)		
1	9 (28)	4 (13)			12 (38)	1 (3)		
Visible vocal cords^*^	23 (72)	28 (88)	0.82 (0.64 to 1.06)	0.213^§^	20 (63)	31 (97)	0.65 (0.49 to 0.85)	0.001^§^

Data are shown as mean ± standard deviation, number (%), or median (Q1, Q3).

* Visible vocal cords were defined as a fiberoptic score of 2–4.

† Mean difference and its 95% confidence interval are shown.

‡Welch’s two-sample t-test was used for comparison.

§Fisher’s exact test was used for comparison.

^II^Mann-Whitney U test was used for comparison.

CI: confidence interval, OLP: oropharyngeal leak pressure

## Discussion

To our knowledge, this is the first study to evaluate the clinical performance of the i-gel® Plus in an East Asian population. The OLP of i-gel® Plus was much lower than that reported in a recent European study [16]. Nevertheless, i-gel® Plus achieved a high first-attempt success rate of 92% and an overall success rate of 98%. Exploratory comparisons by sex suggested lower OLP and fewer visible vocal cords on fiberoptic examination in male patients than in female patients.

In this study, the overall OLP was 23.7 ± 6.9 cmH_2_O in Japanese patients, which was much lower than the 32 cmH_2_O reported by Werner et al [16]. In this cohort, the overall OLP was unlikely to have been lowered solely by inappropriate placement in some patients, given that the Q-Q plot showed an approximately normal distribution. Moreover, this study included only anesthesiologists with sufficient experience using conventional i-gel® who had also completed i-gel® Plus simulation training. Although one case was converted to tracheal intubation because of poor fit, the overall success rate of 98% was similar to those reported in previous large-scale studies on i-gel® and i-gel® Plus studies [4,16]. Therefore, technical inexperience alone is unlikely to fully explain the lower OLP observed in the present study.

i-gel® was designed based on hypopharyngeal and laryngeal anatomy, unlike other SGAs, and was subsequently evaluated anatomically in the UK and USA [1,2]. Despite the unclear details on i-gel® Plus development, the lower OLP observed in this study may partly reflect anatomical differences between East Asian and Western populations [17], although this remains speculative because no direct anatomical measurements or imaging data were obtained. When positioned correctly, the proximal part of the i-gel® bowl can lift the epiglottis, allowing only the vocal cords to be seen through a fiberscope [1]. In the European study by Werner et al., 10% of the patients did not have visible vocal cords [16]; however, in the present study, 20% did not have visible vocal cords. These findings from fiberoptic examination and OLP measurement suggest that i-gel® Plus may not have been optimally positioned in some patients.

The clinical performance of i-gel® has been evaluated [2,13–15,22,25]. Despite its anatomy-based design, population-related anatomical variation has received limited attention in the evaluation of SGAs. Similar issues have been discussed for other anatomy-based medical devices, such as artificial joints [26,27]. Such variation may warrant consideration when evaluating devices developed on the basis of anatomical structures.

Moreover, appropriate size selection must be addressed. In a multicenter study conducted in France, the sex-based i-gel® size selection (size 5 for male patients and size 4 for female patients) resulted in fewer inadequate initial ventilations than the weight-based selection [20]. In a crossover study of Japanese women weighing 50–60 kg, the OLP was higher with size 3 i-gel® than with size 4 [21]. Based on the literature and our clinical experience, we selected the device size primarily by sex. The manufacturer’s weight-based recommendation does not always determine a unique size because the recommended weight ranges overlap, and the instructions also advise consideration of patient body habitus. Because sealing performance and anatomical positioning may vary according to device size, the sex-based sizing strategy used in this study may have contributed to both the OLP and the fiberoptic findings. Therefore, these findings should be interpreted with consideration of the selected sizing approach. Future studies are needed to determine whether optimizing size selection can improve the fit performance of i-gel® Plus.

In the exploratory comparisons stratified by age and sex, the OLP in male patients was lower than that in female patients; however, the difference was not clinically meaningful. Male sex has been reported as a risk factor for ventilation failure with SGAs [4,19]. In the present study, the observed difference between male and female patients should be interpreted cautiously because the comparisons were exploratory and a slight imbalance in neuromuscular blocking drug use (100% in male patients and 88% in female patients) may also have affected performance. Further studies are needed to clarify whether sex-related anatomical or clinical factors may affect the fit performance of i-gel® Plus.

Except for the OLP, the secondary outcomes were clinically favorable. In this study, the first-attempt success rate was high (92%), which was similar to those of large-scale European studies of i-gel® Plus (88%) and i-gel® (93%) [4,16]. The insertion time was longer than that reported by Werner et al.; however, direct comparison is limited because their definition of insertion time differed from ours. They defined insertion time as the duration from the tip crossing the teeth until the device was connected to the anesthetic machine [16,28], whereas we measured it from picking up the i-gel® Plus until the first upslope appeared in the capnogram, which is a commonly used definition in previous studies [3,5,22]. In studies using the same definition as ours, the insertion time of i-gel® was approximately 20 s [3,5,22]. Thus, the insertion time of i-gel® Plus in our study appears similar to that previously reported for i-gel®. The rates of airway injury, hoarseness, and sore throat were comparable to those reported by Werner et al. [16]. Gastric tube placement was also evaluated, and 94% of cases were rated as easy. The ease of placing a large-bore gastric tube may be an additional practical advantage of i-gel® Plus.

This study had several strengths. First, this study is the first to investigate the clinical performance of i-gel® Plus in Asia. The results raise the possibility that i-gel® Plus performance may differ across populations, although no direct assessment of racial or anatomical differences was performed in this study. Second, fiberoptic observations were performed in all patients. Our findings regarding the relatively poor i-gel® Plus positioning and low OLP may help to explain possible differences in fit performance across populations. Finally, the performance of gastric tube insertion was evaluated. The favorable performance of gastric tube insertion may represent an additional practical advantage of i-gel® Plus over the conventional i-gel®.

This study also had some limitations. First, this was a single-center study with a relatively small sample size and only included Japanese patients. Thus, the results may not be generalizable to other settings, including other Asian populations. Second, this was a single-arm study without a comparator device. Thus, it remains unclear whether the lower OLP observed in this cohort was related to the device itself or to other clinical and procedural factors. Future comparative studies across different populations and with other SGAs are warranted. Third, patients with obesity, predicted difficult airways, and several surgical conditions were excluded. Therefore, the generalizability of our findings is limited, and the results may not be directly applicable to specific clinical scenarios involving patients with obesity, patients with predicted difficult airways, or patients undergoing laparoscopic surgery. Fourth, this study only included experienced anesthesiologists, which may limit the generalizability of our results. Finally, comparisons of i-gel® Plus performance by age and sex were exploratory and not adjusted for multiple comparisons. Therefore, these findings, particularly the differences observed between male and female patients, should be interpreted cautiously.

## Conclusions

In conclusion, i-gel® Plus showed a high first-attempt success rate of 92%; however, the OLP was lower than expected at 23.7 ± 6.9 cmH_2_O in this Japanese cohort. Based on the results of our study, i-gel® Plus may be a feasible option in selected East Asian patients, particularly when placement of a large-bore gastric tube is desired. However, because this was a single-arm study conducted in a selected Japanese population, further comparative studies are needed to confirm the generalizability of our findings, especially for advanced applications such as laparoscopic surgery or use in patients with obesity. Possible population-related anatomical differences warrant further investigation in future studies assessing devices designed according to anatomical structures. Furthermore, future studies on optimal size selection across different populations are warranted.

## Supporting information

S1 Table Detailed background and perioperative characteristics of the patients stratified by age and sex. (DOCX)

S2 Table Detailed background and perioperative characteristics of the patients, 16 patients in each group. (DOCX)

S3 Table Postoperative hoarseness and sore throat after the i-gel® Plus use. (DOCX)

S1 Fig Distribution of oropharyngeal leak pressure of i-gel® Plus across different patient groups. Each colored circle represents the observed value for one patient. The lower and upper edges of the boxes indicate the 25th and 75th percentiles, respectively. The bold horizontal line within each box represents the median. The whiskers extend to the lowest and highest values within 1.5 times the interquartile range. Outliers are shown as circles.
(EPS)

S1 Checklist STROBE-checklist-v4-cohort. (DOC)
