# Supplementary material for: Clinical evaluation of the i-gel Plus supraglottic airway in Japanese patients: A prospective observational study
Source: PLoS One. 2026 May 7;21(5):e0349108. doi: 10.1371/journal.pone.0349108 (PMC13152166; doi:10.1371/journal.pone.0349108)
Supplement: S1 Table — (DOCX) [file pone.0349108.s001.docx]

**S1 Table.** **Detailed background and perioperative characteristics of the patients stratified by age and sex**

| **Variables** | **Total**  **N = 64** | **Nonelderly^*^**  **N = 32** | **Elderly^†^**  **N = 32** | **Male**  **N = 32** | **Female**  **N = 32** |
| --- | --- | --- | --- | --- | --- |
| Age | 69 (55, 77) | 55 (42, 64) | 78 (76, 81) | 69 (56, 76) | 70 (53, 78) |
| Male sex | 32 (50) | 16 (50) | 16 (50) | 32 (100) | 0 (0) |
| Height (cm) | 160.9 (152.0, 167.9) | 163.1 (158.3, 173.3) | 154.7 (150.0, 165.2) | 167.2 (162.4, 174.1) | 152.0 (149.2, 160.9) |
| Weight (kg) | 60.8 (50.9, 68.1) | 61.1 (51.9, 70.7) | 58.9 (50.9, 67.6) | 66.0 (60.0, 73.0) | 53.0 (45.7, 63.0) |
| BMI (kg/m^2^) | 23.2 (20.4, 26.3) | 22.6 (19.6, 25.5) | 23.3 (21.3, 26.5) | 23.3 (22.4, 26.5) | 22.2 (19.3, 25.0) |
| ASA classification  1  2  3  4 | 11 (17)  48 (75)  4 (6)  1 (2) | 11 (34)  21 (66)  0 (0)  0 (0) | 0 (0)  27 (84)  4 (13)  1 (3) | 4 (13)  25 (78)  2 (6)  1 (3) | 7 (22)  23 (72)  2 (6)  0 (0) |
| Mallampati class  1  2  3  Not recorded | 50 (78)  8 (13)  2 (3)  4 (6) | 24 (75)  4 (13)  1 (3)  3 (9) | 26 (81)  4 (13)  1 (3)  1 (3) | 27 (84)  3 (9)  2 (6)  0 (0) | 23 (72)  5 (16)  0 (0)  4 (13) |
| Restricted neck movement  Mild | 4 (6) | 2 (6) | 2 (6) | 3 (9) | 1 (3) |
| Asthma | 1 (2) | 1 (3) | 0 (0) | 1 (3) | 0 (0) |
| Hypertension | 31 (48) | 6 (19) | 25 (78) | 14 (44) | 17 (53) |
| Diabetes mellitus | 6 (9) | 1 (3) | 5 (16) | 3 (9) | 3 (9) |
| Dyslipidemia | 17 (27) | 4 (13) | 13 (41) | 8 (25) | 9 (28) |
| Ischemic heart disease | 1 (2) | 0 (0) | 1 (3) | 1 (3) | 0 (0) |
| COPD | 0 (0) | 0 (0) | 0 (0) | 0 (0) | 0 (0) |
| Smoking  Never  Past  Current | 36 (56)  19 (30)  9 (14) | 19 (59)  7 (22)  6 (19) | 17 (53)  12 (38)  3 (9) | 9 (28)  14 (44)  9 (28) | 27 (84)  5 (16)  0 (0) |
| Surgery type  Orthopedic  Urologic  Mammectomy  Gynecologic  General  Dermatologic | 20 (31)  20 (31)  14 (22)  5 (8)  4 (6)  1 (2) | 13 (41)  9 (28)  6 (19)  4 (13)  0 (0)  0 (0) | 7 (22)  11 (34)  8 (25)  1 (3)  4 (13)  1 (3) | 11 (34)  17 (53)  0 (0)  0 (0)  3 (9)  1 (3) | 9 (28)  3 (9)  14 (44)  5 (16)  1 (3)  0 (0) |
| Surgery time (min) | 69.0 (53.3, 97.3) | 68.0 (48.5, 101.8) | 71.5 (58.8, 92.3) | 77.5 (64.8, 108.5) | 60.5 (47.8, 86.8) |
| Anesthesia time (min) | 116.0 (96.5, 141.0) | 116.5 (93.5, 145.0) | 116.0 (100.8, 136.2) | 122.0 (104.5, 149.2) | 102.5 (92.8, 132.0) |
| Induction anesthetics  Propofol  Remimazolam | 60 (94)  4 (6) | 28 (88)  4 (13) | 32 (100)  0 (0) | 29 (91)  3 (9) | 31 (97)  1 (3) |
| Neuromuscular blocking drugs | 60 (94) | 28 (88) | 32 (100) | 32 (100) | 28 (88) |
| Total intravenous anesthesia | 45 (70) | 27 (84) | 18 (56) | 16 (50) | 29 (91) |
| Adjuvant analgesics  Flurbiprofen  Acetaminophen  Dexamethasone  Other steroids | 30 (47)  59 (92)  45 (70)  13 (20) | 17 (53)  31 (97)  23 (72)  6 (19) | 13 (41)  28 (88)  22 (69)  7 (22) | 10 (31)  30 (94)  20 (63)  6 (19) | 20 (63)  29 (91)  25 (78)  7 (22) |
| Size of i-gel® Plus  3  4 | 31 (48)  33 (52) | 16 (50)  16 (50) | 15 (47)  17 (53) | 0 (0)  16 (100) | 31 (97)  1 (3) |
| Anesthesiologists with ≥ 2-year experience | 51 (80) | 23 (72) | 29 (91) | 24 (75) | 28 (88) |

The data are shown as median (Q1, Q3) or number (%).

^*^Nonelderly patients were defined as age < 70 years.

^†^Elderly patients were defined as age ≥ 70 years.

ASA: American Society of Anesthesiologists, BMI: body mass index, COPD: chronic obstructive pulmonary disease
