# Supplementary material for: Clinical evaluation of the i-gel Plus supraglottic airway in Japanese patients: A prospective observational study
Source: PLoS One. 2026 May 7;21(5):e0349108. doi: 10.1371/journal.pone.0349108 (PMC13152166; doi:10.1371/journal.pone.0349108)
Supplement: S2 Table — (DOCX) [file pone.0349108.s002.docx]

**S2 Table.** **Detailed background and perioperative characteristics of the patients, 16 patients in each group**

| **Variables** | **Nonelderly^*^ male**  **N = 16** | **Nonelderly^*^ female**  **N = 16** | **Elderly^†^ male**  **N = 16** | **Elderly^†^ female**  **N = 16** |
| --- | --- | --- | --- | --- |
| Age | 56 (46, 64) | 51 (42, 65) | 77 (75, 82) | 79 (77, 81) |
| Male sex | 16 (100) | 0 (0) | 16 (100) | 0 (0) |
| Height (cm) | 173.6 (163.2, 176.3) | 159.3 (153.9, 163.1) | 165.5 (162.1, 167.5) | 150.0 (147.5, 152.0) |
| Weight (kg) | 70.8 (65.3, 80.0) | 50.9 (45.6, 56.4) | 61.0 (56.0, 68.3) | 53.4 (48.5, 67.1) |
| BMI (kg/m^2^) | 24.4 (22.7, 28.2) | 20.0 (18.7, 22.6) | 23.2 (20.7, 23.6) | 24.4 (22.1, 28.7) |
| ASA classification  1  2  3  4 | 4 (25)  12 (75)  0 (0)  0 (0) | 7 (44)  9 (56)  0 (0)  0 (0) | 0 (0)  13 (81)  2 (13)  1 (6) | 0 (0)  14 (88)  2 (13)  0 (0) |
| Mallampati class  1  2  3  Not recorded | 13 (81)  2 (13)  1 (6)  0 (0) | 11 (69)  2 (13)  0 (0)  3 (19) | 14 (88)  1 (6)  1 (6)  0 (0) | 12 (75)  3 (19)  0 (0)  1 (6) |
| Restricted neck movement  Mild | 1 (6) | 1 (6) | 2 (13) | 0 (0) |
| Asthma | 1 (6) | 0 (0) | 0 (0) | 0 (0) |
| Hypertension | 2 (13) | 4 (25) | 12 (75) | 13 (81) |
| Diabetes mellitus | 1 (6) | 0 (0) | 2 (13) | 3 (19) |
| Dyslipidemia | 2 (13) | 2 (13) | 6 (38) | 7 (44) |
| Ischemic heart disease | 0 (0) | 0 (0) | 1 (6) | 0 (0) |
| COPD | 0 (0) | 0 (0) | 0 (0) | 0 (0) |
| Smoking  Never  Past  Current | 6 (38)  4 (25)  6 (38) | 13 (81)  3 (19)  0 (0) | 3 (19)  10 (63)  3 (19) | 14 (88)  2 (13)  0 (0) |
| Surgery type  Orthopedic  Urologic  Mammectomy  Gynecologic  General  Dermatologic | 8 (50)  8 (50)  0 (0)  0 (0)  0 (0)  0 (0) | 5 (31)  1 (6)  6 (38)  4 (25)  0 (0)  0 (0) | 3 (19)  9 (56)  0 (0)  0 (0)  3 (19)  1 (6) | 4 (25)  2 (13)  8 (50)  1 (6)  1 (6)  0 (0) |
| Surgery time (min) | 87.0 (65.8, 98.3) | 55.0 (44.0, 86.0) | 74.0 (64.8, 95.8) | 66.5 (51.0, 87.8) |
| Anesthesia time (min) | 125.0 (98.5, 154.0) | 100.5 (85.3, 131.8) | 119.5 (105.8, 141.5) | 105.0 (97.5, 132.0) |
| Induction anesthetics  Propofol  Remimazolam | 16 (100)  0 (0) | 16 (100)  0 (0) | 13 (81)  3 (19) | 15 (94)  1 (6) |
| Neuromuscular blocking drugs | 16 (100) | 12 (75) | 16 (100) | 16 (100) |
| Total intravenous anesthesia | 11 (69) | 16 (100) | 5 (31) | 13 (81) |
| Adjuvant analgesics  Flurbiprofen  Acetaminophen  Dexamethasone  Other steroids | 7 (44)  15 (94)  10 (63)  3 (19) | 10 (63)  16 (100)  13 (81)  3 (19) | 3 (19)  15 (94)  10 (63)  3 (19) | 10 (63)  13 (81)  12 (75)  4 (25) |
| Size of i-gel® Plus  3  4 | 0 (0)  16 (100) | 16 (100)  0 (0) | 0 (0)  16 (100) | 15 (94)  1 (6) |
| Anesthesiologists with ≥ 2-year experience | 11 (69) | 12 (75) | 13 (81) | 16 (100) |

The data are shown as median (Q1, Q3) or number (%).

^*^Nonelderly patients were defined as age < 70 years.

^†^Elderly patients were defined as age ≥ 70 years.

ASA: American Society of Anesthesiologists, BMI: body mass index, COPD: chronic obstructive pulmonary disease
