# Supplementary material for: Clinical evaluation of the i-gel Plus supraglottic airway in Japanese patients: A prospective observational study
Source: PLoS One. 2026 May 7;21(5):e0349108. doi: 10.1371/journal.pone.0349108 (PMC13152166; doi:10.1371/journal.pone.0349108)
Supplement: S3 Table — (DOCX) [file pone.0349108.s003.docx]

**S3 Table** Postoperative hoarseness and sore throat after the i-gel® Plus use

|  | **Postanesthesia care unit**  **N = 64** | **Postoperative day 1**  **N = 64** |
| --- | --- | --- |
| **Hoarseness** |  |  |
| None | 52 (81) | 59 (92) |
| Mild | 7 (11) | 4 (6) |
| Moderate | 4 (6) | 1 (2) |
| Severe | 0 (0) | 0 (0) |
| Not recorded | 1 (2)^*^ | 0 (0) |
| **Sore throat** |  |  |
| None | 57 (89) | 57 (89) |
| Mild | 6 (9) | 7 (11) |
| Moderate | 0 (0) | 0 (0) |
| Severe | 0 (0) | 0 (0) |
| Not recorded | 1 (2)^*^ | 0 (0) |

The data are shown as number (%).

^*^One patient could not express severity of their throat symptoms at the postanesthesia care unit.
